# Intermittent oral levetiracetam reduced recurrence of febrile seizure accompanied with epileptiform discharge: a pilot study

**DOI:** 10.1186/s13052-018-0507-8

**Published:** 2018-06-15

**Authors:** Lin-Yan Hu, Xiu-Yu Shi, Hui Li, Meng-Na Zhang, Shu-Fang Ma, Li-Ping Zou

**Affiliations:** Department of Pediatrics, Chinese PLA General Hospital, Beijing, 100853 China

**Keywords:** Febrile seizure, Children, Electroencephalogram, Epileptiform discharge, Levetiracetam

## Abstract

**Background:**

In previous study, we have found intermittent oral levetiracetam (LEV) can effectively prevent recurrence of febrile seizure (FS). This study aimed to analyze the effects of the preventive on the patients with frequent FS accompanied with epileptiform discharge.

**Methods:**

Patients with frequent FS were assigned to undergo Electroencephalogram (EEG). At the onset of fever, the patients who presented epileptiform discharge were orally administered with LEV with a dose of 15–30 mg/kg per day twice daily for 1 week, thereafter, the dosage was gradually reduced until totally discontinued in the second week. The seizure frequency associated with febrile events and FS recurrence rate during a 48-week follow-up were analyzed.

**Results:**

among the 19 patients presented epileptiform discharge on EEG, 31.58% (6 of 19) had complex FS, 68.42% (13 of 19) had simple FS. Up to 57.89% (11 of 19) had a family history of seizure disorder and 36.84% (7 of 19) had a family history of FS in first-degree relatives. 42.11% (8 of 19) happened the first FS episode at the age < 18 months. 36.84% (7/19) presented generalized spikes, 63.16% (12/19) showed focal spikes. During the 48-week follow-up period, the patients experienced 26 febrile episodes, none of them presented seizure recurrence.

**Conclusion:**

Intermittent oral LEV can prevent the seizure recurrence of FS accompanied with epileptiform discharge in 48-week. However, further randomized controlled trials should be conducted.

**Trial registration:**

ChiCTR-IPR-15007241; Registered 1 January 2014 - Retrospectively registered.

## Background

Approximately 8% of people will experience at least one seizure episode during their lifetime [1]. Up to 30% of such episodes are febrile seizures (FS), which are the most commonly occurring seizures in 2 to 5% of all children. FS is a benign condition. Children who have or have not suffered FS before their fifth birthday can attain similar academic and social successes [2]. Nevertheless, although FS is a benign condition in most cases and the prognosis is good generally and recurrences do not impair the prognosis in children who were neurologically normal before their first febrile seizure, FS episodes constitute a traumatic experience, it is a very frightening event for the parents/caregivers witnessing a tonic–clonic seizure, especially for the patients with frequent FS, they suffer extreme anxiety for recurrences of seizures or development of epilepsy; FS is also likely one of the most frequent causes of admittance to pediatric emergency wards worldwide [3]. In any case, febrile seizures should be taken under serious consideration. In previous years, interest has increased considerably in preventing FS and reducing its recurrence risk either by continuous treatment with antiepileptic drugs (AEDs) such as phenobarbital and valproic acid (VPA) or with intermittent treatment with a drug such as diazepam. However, although phenobarbital, VPA, and primidone are considered effective in preventing the recurrence of FS when continuously administered [4], long-term treatment with such drugs is associated with a wide spectrum of adverse effects, including sedation, behavioral changes, gastrointestinal and hematologic toxicity, hypersensitivity reactions, and rare fatal hepatotoxicity with VPA in young children. And also the intermittent administration of benzodiazepines (e.g., diazepam and midazolam) at the onset of fever is effective [5, 6], but the effectiveness of this treatment is limited because sedative effects can mask the signs and symptoms of any evolving central nervous system infection [4, 7, 8]. Considering that the potential toxicities associated with antiepileptic therapy outweigh the relatively minor risks associated with FS, the American Academy of Pediatrics does not recommend continuous antiepileptic therapy with phenobarbital or VPA and intermittent therapy with diazepam to prevent FS recurrences [4, 9]. Then, during 2009 to 2011, in order to find a safe and effective therapy to prevent FS recurrences, we performed a multicenter, randomized, controlled, 48-week follow-up parallel-group outpatient study in children with FS, and verified that intermittent oral LEV can help the patients with frequent FS to reduce the recurrence of FS effectively and safely [10].

Patients with repeated recurrences of FS who presented epileptiform discharge on EEG were always considered to be prone to epilepsy; thus, some of these patients accepted continuous treatment with antiepileptic drugs, such as valproic acid and topiramate, during their follow-up period, in which few patients experienced seizure recurrence [11]. However, long-term treatment with such drugs can contribute to the child’s distress and is associated with a wide avoidable spectrum of adverse effects, while also consuming medical resources. Actually, simple, effective, and safe method for prevention of recurrent FS would be desirable for the patients with frequent FS who presented epileptiform discharge. In our previous study, we have identified LEV was effective in individuals with electrical status epilepticus during sleep (ESES) also because it can ameliorate the abnormal EEG [12]. Here, we summarized the characteristics of the patients with frequent FS accompanied with epileptiform discharge and analyzed the effects of intermittent oral LEV on reducing the recurrence of FS for these patients.

## Methods

### Patients and study design

We performed a one-center, 48-week follow-up outpatient pilot study in children with FS. The criteria for inclusion were as follows: children with a history of two or more episodes of FS within the last 6 months, at least one seizure recurrence within the last 2 weeks, and onset age between 3 months and 5 years. The participants were recruited from 1 January 2014 to 1 January 2015. Another round of selection was performed in accordance with the further assessment of their conditions. The criteria for exclusion were as follows: episodes of previous seizures without fever, intracranial infections or head trauma, or current use of AEDs. The criteria for diagnosis of complex FS were as follows: FS duration longer than 15 min, repeated convulsions within the same day, and focal seizure activity or focal findings during the postictal period. Patients who conformed to criteria were assigned to undergo EEG. Each EEG was normally recorded with a 32 channel digital machine for ≥30 min in the sleep and waking states more than 2 weeks after the latest FS episode. All EEGs were read by one experienced pediatric neurologist and one electroencephalographer. Only focal spikes, sharp waves, or generalized spikes and waves were included as epileptiform discharges. The foci of epileptiform discharges were classified into five regions: frontal, central, parietal, temporal, and occipital areas. Unprovoked seizures were defined as those with no immediately recognizable stimulus or cause. Epilepsy was defined as at least two unprovoked (or reflex) seizures occurring > 24 h apart [13].

All FS patients who showed epileptiform discharges accepted intermittent oral LEV to prevent recurrences. The procedure was described in previous study [10], simply put, parents/caregivers were instructed to take a child’s temperature immediately when the child appears ill or feverish, such as in cases of runny nose or nasal obstruction, hot flashes, sore throat, and constipation. Parents/caregivers were also instructed to administer promptly the study medication when the temperature indicates a fever. Patients in the LEV group received oral LEV at a dose of 15–30 mg/kg per day twice daily at the onset of fever (*T* > 37.5 °C) for 1 week (therapy period), followed by dose tapering of 50% every 2 days until complete withdrawal at the second week (decrement period). The parent/caregiver was instructed to administer any other antipyretic drug to their child when *T* > 38.5 °C, with or without antibiotics as deemed appropriate by the attending pediatrician.

In accordance with the protocols, birth and development history, FS and seizure family history, liver and kidney function, FS frequency before enrollment, FS onset age were taken. Pediatric neurology examination was performed for all patients. Parents/caregivers were fully informed about the nature and management of FS. Follow-up observation tables were also distributed to the parents/caregivers for the recording of febrile and seizure events, and adverse effects after medication at home. Parents/caregivers were contacted by telephone every 12 weeks to reinforce the study program. At each episode of febrile illness, parents/caregivers called the doctor and provided all the necessary information about recurrence. The primary variable in efficacy was seizure frequency associated with febrile events and FS recurrence rate (RR) during the 48-week follow-up. The second variable in efficacy was the side effects associated with the drugs. These variables were analyzed at the end of the evaluation period. For tolerability assessment, vital signs were assessed by multiparameter patient monitoring, including conditions such as mental state (e.g., changes in temper, lethargy), gastrointestinal symptoms (e.g., poor appetite, stomachache, vomiting), skin-related changes (e.g., rashes, pruritus), and body temperature observed at home. Upon admission to the study, parents/caregivers were instructed on what and how to observe vital signs. The parents/caregivers were assigned to assess treatment tolerability in accordance with the designed follow-up observation table. We measured the primary and secondary endpoints after all follow-up information on the patients were collected.

### Statistical analysis

All available data were used. Patients who discontinued the treatment early completed all end-of-study assessments. Descriptive statistics were used in the analysis of all efficacy, demographic, and baseline variables. Chi-square and Fisher’s exact test were used to analyze the constituent ratio of gender, family convulsion history, FS type, onset age, visiting age, course of disease, and FS frequency before enrollment, the data was described with the median (Q1–Q3). The Wilcoxon rank-sum test was applied to compare the differences in these indexes between the simple FS and complex FS. All statistical analyses were conducted by using SPSS 21.0 (SPSS Inc., Chicago, IL), and *P* < 0.05 was considered statistically significant.

## Results

**Fig. 1:**
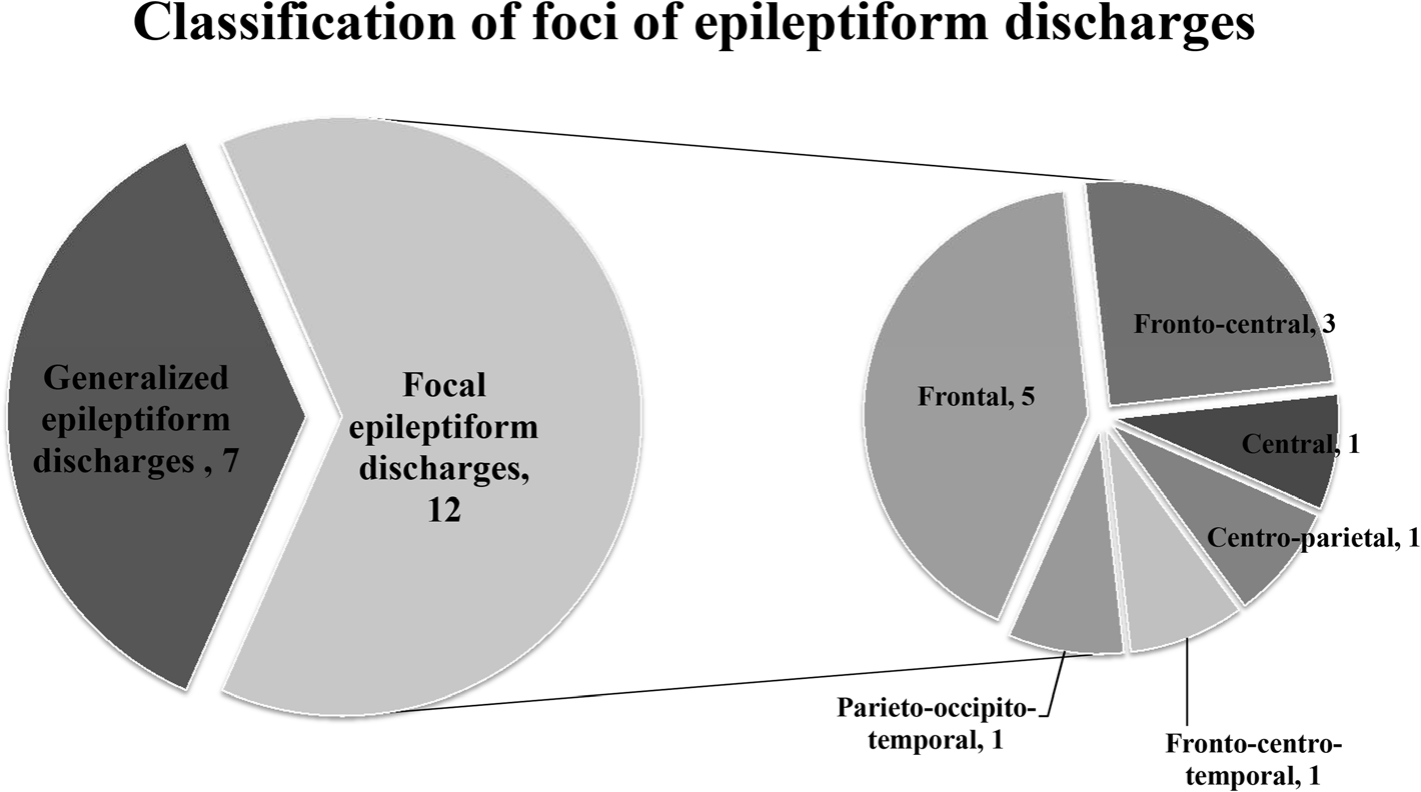
Classification of foci of epileptiform discharges: Among the 19 patients with epileptiform discharges on EEG, 7 (36.84%) presented generalized spikes, and 12 (63.16%) showed focal spikes, of which 5 patients (26.32%) had epileptiform discharges from the frontal area, 3 (15.79%) from the fronto-central area, 1 (5.26%) from the central area, 1 (5.26%) from the centro-parietal area, 1 (5.26%) from the fronto-centro-temporal area, and 1 (5.26%) from the parieto-occipito-temporal area

**Fig. 2:**
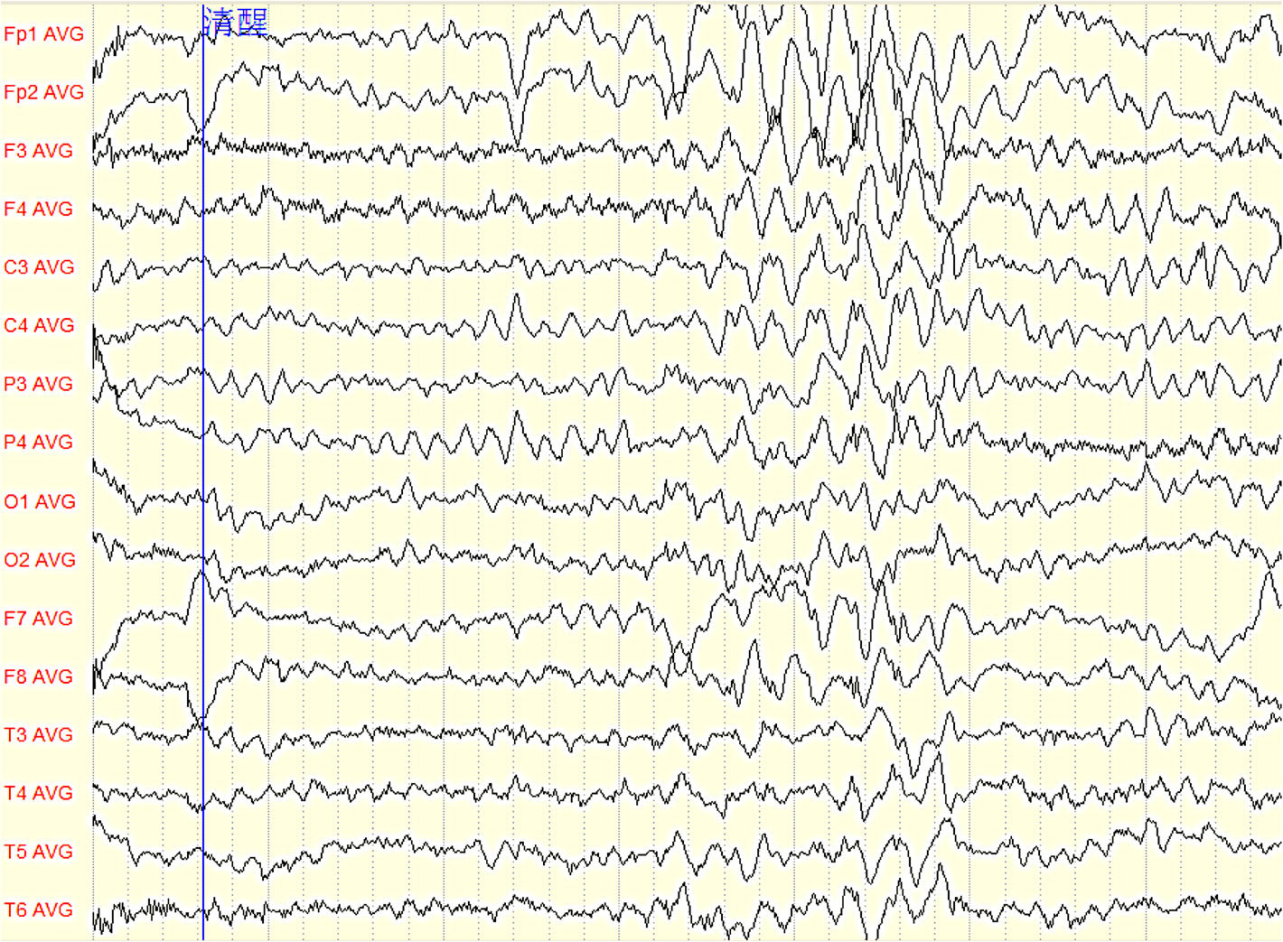
The EEG characteristic of one patient: paroxysmal 3-4 Hz spike-and-waves, slow waves were presented in all leads, especially in frontal area

The ranges of FS onset age and FS course in the group with abnormal EEG were 3–52, 3–51 months, respectively, for all the 19 children (15 males and 4 females) with FS, who presented epileptiform discharges on EEG. Correspondingly, the medians (Q1–Q3) were 19 (11–27) and 17 (8–36). The range of FS frequency before enrollment was 2–10 times, and the median (Q1–Q3) was 3 (3–5). Among the patients, 42.11% (8 of 19) happened the first FS episode at the age < 18 months; 57.89% (11 of 19) had a family history of seizure disorder and 36.84% (7 of 19) had a family history of FS in first-degree relatives. Among all the 19 patients, 68.42% (13 of 19) had simple FS, of which 6 had a family history of seizure disorder. Up to 31.58% (6 of 19) of all the patients had complex FS, whereas 5 had a family history of seizure disorder. Among the 19 patients with epileptiform discharges on EEG, 7 (36.84%) presented generalized spikes, and 12 (63.16%) showed focal spikes, of which 5 patients (26.32%) had epileptiform discharges from the frontal area, 3 (15.79%) from the fronto-central area, 1 (5.26%) from the central area, 1 (5.26%) from the centro-parietal area, 1 (5.26%) from the fronto-centro-temporal area, and 1 (5.26%) from the parieto-occipito-temporal area (Figs. 1 and 2).

In the patients with simple FS, the ranges of FS onset age and FS course were 3–37, 3–51, respectively, the medians (Q1–Q3) were 19(13.5–24.5), 12 (5–26), respectively. The range of FS frequency before enrollment was 2–12 times, the median (Q1–Q3) was 3 (2.5–4.5). Among the sample, 46.15% (six of 13) had a family history of seizure disorder, 62.5% (five of 8) happened the first FS episode at the age < 18 months, 76.92% (ten of 13) showed focal spikes. While in the patients with complex FS, the ranges of FS onset age and FS course were 5.5–52, 8–50, respectively. The medians (Q1–Q3) were 17.5 (8.9–37.8), 34.5 (9.5–47.4), respectively. The range of FS frequency before enrollment was 3–7 times. The median (Q1–Q3) was 4(3–6.3). Among the sample, 83.33% (five of 6) had a family history of seizure disorder, 50.0% (three of 6) happened the first FS episode at the age < 18 months, 50% (two of 4) showed focal spikes. No significant differences were found in gender constitution (male/female), onset age, disease cause, and FS frequency before enrollment, epileptiform discharges on EEG (focal/generalized), patients with family convulsion history between the two groups (*P* > 0.05). Tables 1, 2 summarize the demographics and characteristics of the sample.

**Table 1:** Demographics and characteristics of the children with FS accompanied with abnormal EEG

	Gender	Visiting age, (months)	FS frequency before enrollment	Onset age, (months)	Type of seizures	Presentation of seizure	Classification of foci of epileptiform discharges	Family convulsion history	Lost	Compliance	Febrile episode	Seizure episode	Developed epilepsy
1	male	31	5	19	simple	\	Fronto-central area	Grandmother with FS	No	Yes	3	0	No
2	male	20	2	15	simple	\	Fronto-central area	No	No	Yes	0	0	No
3	male	40	4	18	simple	\	Frontal area	Mother and uncle with FS	Yes	Yes	\	\	\
4	male	54	3	3	simple	\	Frontal area	No	No	Yes	0	0	No
5	male	28	6	11	simple	\	Fronto-centro-temporal area	No	No	Yes	0	0	No
6	male	19	3	16	simple	\	Fronto-central area	Mother with FS	No	Yes	3	0	No
7	male	19	5	11	complex	Duration longer than 15 min	Generalized spikes	Father and mother with FS	No	Yes	3	0	No
8	female	43	3	33	complex	Repeated convulsions within 24 h; Duration longer than 40 min	Generalized spikes	No	Yes	Yes	\	\	\
9	male	48	4	12	simple	\	Frontal area	Uncle with FS	No	Yes	1	0	No
10	male	52	3	5.5	complex	Focal seizure activity; Duration longer than 30 min	Frontal area	Father with FS	Yes	Yes	\	\	\
11	female	32	2	27	simple	\	Generalized spikes	No	No	Yes	1	0	No
12	female	60	7	10	complex	Duration longer than 15 min	Generalized spikes	Father with FS	No	Yes	2	0	No
13	male	32	3	22	simple	\	Central area	Uncle with FS	No	Yes	1	0	No
14	female	53	10	29	simple	\	Generalized spikes	No	No	Yes	3	0	No
15	male	48	3	19	simple	\	Frontal area	Mother with FS	No	Yes	2	0	No
16	male	76	3	52	complex	Focal seizure activity	Generalized spikes	Mother with FS	No	Yes	1	0	No
17	male	49	4	37	simple	\	Generalized spikes	No	No	Yes	0	0	No
18	male	69	6	24	complex	Repeated convulsions within 24 h	Parieto-occipito-temporal area	Cousin with epilepsy	No	Yes	2	0	No
19	male	24	2	20	simple	\	Centro-parietal area	No	No	Yes	4	0	No

**Table 2:** Demographics and characteristics of the enrolled children

Variable	Total *N* = 19	Simple *N* = 13	Complicated *N* = 6	Values	*P* value (two-sided)
Gender (No.)					
Male	15	11	4		
Female	4	2	2	0.796^1^	0.557^2^
Onset age (months)					
Range	3–52	3–37	5.5–52		
M (Q1–Q3)	19(11–27)	19(13.5–24.5)	17.5(8.9–37.8)	-0.044^3^	0.965^4^
Onset age (No.)					
< 18 months	8	5	3		
> 18 months	11	8	3	0.224^1^	1.000^2^
Course of disease (months)					
Range	3–51	3–51	8–50		
M (Q1–Q3)	17(8–36)	12(5–26)	34.5(9.5–47.4)	-1.318^3^	0.188^4^
FS frequency before enrollment (No.)					
Range	2–10	2–10	3–7		
M (Q1–Q3)	3(3–5)	3(2.5–4.5)	4(3–6.3)	-0.904^3^	0.366^4^
Epileptiform discharges on EEG (No.)					
Focal	12	10	2		
Generalized	7	3	4	3.352^1^	0.129^2^
Family convulsion history (No.)					
Yes	11	6	5		
No	8	7	1	2.328^1^	0.177^2^

^1^χ^2^ value

^2^Fisher exact test

^3^Z value

^4^Wilcoxon rank-sums test

During the 48-week follow-up, 3 patients lost to follow up, none of the participants discontinued participation because of diagnosed epilepsy, and all the patients were compliant, the left 16 children experienced up to 26 febrile episodes, four children had no febrile episodes, none experienced seizure recurrence. On the basis of the reports of the parents/caregivers of children who took the study medication during the course of fever, only one child with simple FS experienced severe drowsiness after taking LEV once. Aside from this case, no other side effects were observed in the patients.

## Discussion

Although EEG may be useful for evaluating patients with complex/atypical features or other risk factors for later epilepsy development, EEG is not indicated following a simple FS. So, no exact incidence rate of EEG abnormalities in children with FS has been reported to date, patients who develop FS occasionally undergo EEG because of frequent repetition of FS, physician’s recommendation, or parental demand; the reported incidence rate of EEG abnormalities varied from 2 to 86% [14]. The number of previous FS episodes was associated with an increasing rate of EEG abnormality, from 18% in children with no previous seizures to 63% in those with four or more previous seizures [15]. The greatest concern for children with recurrent FS episodes is the possibility of subsequent FS and unprovoked seizures or epilepsy. In Wo’s retrospective study on the correlation between epileptiform discharges upon EEGs after FS and the prognosis of patients in terms of the development of epilepsy and recurrence of FS, 25.0% patients in the abnormal EEG group showed a statistically significant risk of epilepsy development compared with 2.3% patients in the control group. The recurrence of FS in the abnormal EEG group (33.3%) was higher than that in the control group (26.4%) [14]. However, opposite opinion was shown in other studies also [16]. Thus, no consistent evidence can confirm that abnormal EEGs are predictive of either the risk of FS recurrence or the development of epilepsy. However, with regard to EEGs, focal epileptiform discharges were identified as risk factors for subsequent epilepsy [17]. Frontal EEG paroxysms were found to be significantly associated with a higher risk for development of epilepsy than paroxysm in other regions of EEG foci [18, 19]. In this study, 47.37% patients (9 out of 19) had epileptiform discharges in the frontal area or area associated with frontal, moreover, for the classification of FS, complex FS episodes were reported in 31.58% patients (6 out of 19), none of them developed epilepsy during the 48-week follow-up. Nevertheless, we want to point out: As so far, the criteria for diagnosis of complex FS were FS duration longer than 15 min, repeated convulsions within the same day, and focal seizure activity or focal findings during the postictal period. The criteria is based on the presentation of seizure episode, however, as to some patients with focal epileptiform discharges on EEG may present generalized seizure, actually, this kind of seizure belongs to focal seizure. So, we recommend that EEG should be done for all patients with recurrent FS, and focal epileptiform discharges on EEG should be guided into the criteria for diagnosis of complex FS. In our study, 76.92% (ten of 13) patients with simple FS diagnosed according to the criteria for diagnosis of complex FS showed focal spikes, of that, 80% (eight of 10) presented epileptiform discharges in the frontal area or area associated with frontal, which was considered to be associated with subsequent epilepsy. As for these patients, we will pay attention to long-term follow-up.

Approximately 30–40% of children with a first FS will experience a recurrence; and some risk factors, such as: low age at onset of FS (≤18 months), recurrence within the same illness at the initial seizure, focality, positive family history of FS in first-degree relatives, et al., were considered as predictors for FS recurrence [16]. And in the Pavlidou’s study, almost half of the children who recurred presented with two or more recurrences. Of those children, 56% had two recurrences and 44% recurred three or more times. The study showed low age at onset and positive family history of FS are the prognostic factors that could predispose children with already one recurrence to a second or more [20]. In our study, 47.37% (9 of 19) happened the first FS episode at the age ≤ 18 months, 57.89% (11 of 19) had a family history of seizure, and some patients presented focal episode and recurrence within the same illness; However, none of them presented seizure recurrence during the 48-week follow-up.

Overall, the results suggest that intermittent oral LEV preventive can protect patients with frequent FS with abnormal EEG from recurrence of FS and developing epilepsy in short term. Anyway, this is a pilot study, few limitations should be noted. Although we previously identified LEV to be effective in individuals with ESES and in ameliorating abnormal EEG, we do not know if intermittent oral LEV can reduce the epileptiform discharges in patients with FS. Moreover, about 30% (2/6) of patients with complex FS were lost to follow-up, this makes difficult a real comparison between the group with simple FS as compared to the one with complex FS, and the study cohort is small, hence, the results are insufficient to draw a sound conclusion from small-sample studies. The findings should be clarified through further RCTs in the future. For the prognosis of FS with epileptiform discharges upon EEG and treated with intermittent oral LEV, the 48-week follow-up period is relatively short. Nevertheless, long-term follow-up observation should be prioritized.

## Conclusion

Intermittent oral LEV can prevent the seizure recurrence of FS accompanied with epileptiform discharge in 48-week. However, further randomized controlled trials should be conducted.
